# Elevated levels of tissue inhibitor of metalloproteinases (TIMPS) in human hepatocellular carcinomas

**DOI:** 10.1186/1476-5926-2-S1-S51

**Published:** 2004-01-14

**Authors:** Eiji Matsumoto, Harushige Nakatsukasa, Kazuhiro Nouso, Shin-ichiro Nakamura, Mayumi Suzuki, Yoshiyuki Kobayashi, Masayuki Uemura, Syuichiro Sato, Ei-ichiro Yumoto, Junko Yokoyama, Sou Tsuboi, Hironori Tanaka, Yoshiataka Takuma, Tatsuya Fujikawa, Yasushi Shiratori

**Affiliations:** 1 Department of Medicine and Medical Science, Okayama University Graduate School of Medicine and Dentistry, and Seno Hospital of Okayama City, Okayama 700, Japan

## Introduction

Hepatocellular carcinoma (HCC) is one of the most prevalent malignancies in Japan. Invasion and metastasis of HCC are frequently observed during the development of HCCs, and are regulated by matrix metalloproteinases (MMPs) and tissue inhibitor of metalloproteinases (TIMPs). We have previously reported the strong expression of TIMP-1 and TIMP-2, and cellular localization of the transcripts in HCCs [1]. But the mechanisms of invasion and metastasis of HCCs, and particularly the role of TIMPs are still unclear. The purpose of this study was to examine the tissue concentration of the TIMP-1 and to study the potential involvement of TIMP-1 in the development of HCCs.

## Methods

### Patients and Tissue Preparation

A total of 60 HCC nodules that received curative surgical resection were studied. The nodules had received no pretreatment. Clinical backgrounds of the patients are shown in Table 1. The tissue was divided into HCC and non-neoplastic liver. Tissue samples (~100 mg) of HCC and non-neoplastic liver were homogenized in 150 microliters of 0.5 M Tris, 1.5 M NaCl, 50 mM CaCl2, 2 mM sodium azide, pH 7.6. The homogenate was centrifuged at 15000 – g for 15 min at 4 degrees C and the supernatant was stored at -80 degrees C until analysis.

**Table 1:** Characteristics of the Patients Investigated in this Study

		**n**
**Cases**		58
**HCC nodules**	60	
**Age**	46–75 (median 66)	
**Sex**	Male	46
	Female	12
**Background**	Chronic hepatitis	42
	Liver cirrhosis	16
**Viral infection**	hepatitis B virus (HBV)	14
	hepatitis C virus (HCV)	42
	HBV and HCV	2
**Serum AFP (ng/ml, mean – SD)**	766 – 2935	
**Tumor size in diameter (mm, mean – SD)**	31 – 21	

### Assay for tissue TIMP-1 concentration

TIMP-1 concentration in the supernatant of tissue homogenate of HCC and non-neoplastic liver was measured by a Enzyme-Linked Immunosorbent Assay system, using a commercial assay kit (Fuji Chemical Co. Japan), which contains two kinds of monoclonal antibodies against bovine TIMP-1.

### Statistics

Difference between the tissue TIMP-1 concentration of HCC and that of non-neoplastic liver was examined by Student's *t *test. The Mann-Whitney's *U*-test was performed to compare the relationship between tissue TIMP-1 concentration and clinicopathological features.

## Results

### Tissue TIMP-1 concentration

The TIMP-1 concentration in HCC was 520 – 602 (mean – SD) ng/mg protein, and was significantly (p &lt; 0.0001) higher than that in the corresponding non-neoplastic liver (114 – 212).

### Correlation between tissue TIMP-1 concentration and pathological features

The TIMP-1 concentration in HCC was examined in the various conditions of HCC invasion; those include the differentiation grade, the presence or absence of capsular formation, the capsular invasion, and the vascular invasion. These results are shown in Table 2. The TIMP-1 concentration of HCC in poorly differentiated HCC was higher than that in well and moderately differentiated HCC (p &lt; 0.01 and p &lt; 0.02, respectively).

**Table 2:** TIMP-1 concentration and pathological features of HCCs

			**TIMP-1 concentration in**	
	**Category**	**n**	**HCC**	**NN**
**Differentiation grade**	Well	12	177*	55
	Moderate	42	254**	63
	Poor	6	1020***	74
**Capsular formation**	present	48	228	63
	absent	12	564	51
**Capsular invasion**	present	39	244	72
	absent	9	211	45
**Vascular invasion**	present	13	867	63
	absent	47	252	57

* vs ***: p &lt; 0.01 ** vs ***: p &lt; 0.02 TIMP-1 concentrations are expressed as median value. NN: non-neoplastic liver.

### Correlation between tissue TIMP-1 concentration and clinical features

The TIMP-1 concentration in HCC was examined in the various conditions of clinical features, that include the age of the patients, the underlying liver (liver cirrhosis or chronic hepatitis), the type of viral infection (hepatitis B virus or hepatitis C virus), serum alpha fetoprotein (AFP) levels, and tumor size. The TIMP-1 concentration in liver cirrhosis was higher than that in chronic hepatitis (p &lt; 0.01).

## Discussion

The present study demonstrated that tissue TIMP-1 concentration in HCCs was significantly higher than that in non-neoplastic liver and that the TIMP-1 concentration in poorly differentiated HCCs was significantly higher than that of well and moderately differentiated HCCs. We have previously reported by using in situ hybridization that the expression of TIMP-1 in HCCs was stronger than that in non-neoplastic liver [1]. The present study further confirms that TIMP-1 is up-regulated in HCCs not only in the transcription but also in the translation level. The primary biological function of TIMPs is the inhibition of MMPs. When the cancer invades the surrounding non-neoplastic tissue, MMPs may play a major role to degrade extracellular matrices. TIMPs play an important role in the regulation of the action of MMPs. But, it has been reported that tissue TIMP-1 concentration in gastric cancer was associated with various pathological factors, and patients with high tissue TIMP-1 concentration had poor prognosis in comparison to those with low TIMP-1 level [2]. The elevated TIMP-1 level was also associated with poor prognosis in lung cancer [3]. It is intriguing that the TIMP-1 concentration in poorly differentiated HCCs was significantly higher than that of well and moderately differentiated HCCs. Our previous studies on mRNA level have not shown the similar results to the present study on the protein level. Generally, the lower the differentiation grade of HCCs, the stronger potential of growth, invasion and metastasis. TIMP-1 has been reported to be a new cell-growth factor [4] and also an anti-angiogenic factor [5]. These lines of evidence may be associated with the reason why the TIMP-1 concentration in poorly differentiated HCCs was high. Further studies on the functional role of TIMPs in HCCs are needed.
